# Alpha-Tocopheryl Succinate Inhibits Autophagic Survival of Prostate Cancer Cells Induced by Vitamin K3 and Ascorbate to Trigger Cell Death

**DOI:** 10.1371/journal.pone.0052263

**Published:** 2012-12-18

**Authors:** Marco Tomasetti, Linda Nocchi, Jiri Neuzil, Jacob Goodwin, Maria Nguyen, Lanfeng Dong, Nicola Manzella, Sara Staffolani, Claudio Milanese, Beatrice Garrone, Renata Alleva, Battista Borghi, Lory Santarelli, Roberto Guerrieri

**Affiliations:** 1 Department of Molecular and Clinical Sciences, Polytechnic University of Marche, Ancona, Italy; 2 Apoptosis Research Group, School of Medical Science and Griffith Health Institute, Griffith University, Southport, Queensland, Australia; 3 Molecular Therapy Group, Institute of Biotechnology, Academy of Sciences of the Czech Republic, Prague, Czech Republic; 4 Angelini Research, S. Palomba-Pomezia, Rome, Italy; 5 Department of Anesthesiology Research Unit, IRCCS Orthopaedic Institute Rizzoli, Bologna, Italy; 6 ARCES, University of Bologna, Bologna, Italy; Univ of Bradford, United Kingdom

## Abstract

**Background:**

The redox-silent vitamin E analog α-tocopheryl succinate (α-TOS) was found to synergistically cooperate with vitamin K3 (VK3) plus ascorbic acid (AA) in the induction of cancer cell-selective apoptosis via a caspase-independent pathway. Here we investigated the molecular mechanism(s) underlying cell death induced in prostate cancer cells by α-TOS, VK3 and AA, and the potential use of targeted drug combination in the treatment of prostate cancer.

**Methodology/Principal Findings:**

The generation of ROS, cellular response to oxidative stress, and autophagy were investigated in PC3 prostate cancer cells by using drugs at sub-toxic doses. We evaluated whether PARP1-mediated apoptosis-inducing factor (AIF) release plays a role in apoptosis induced by the combination of the agents. Next, the effect of the combination of α-TOS, VK3 and AA on tumor growth was examined in nude mice. VK3 plus AA induced early ROS formation associated with induction of autophagy in response to oxidative stress, which was reduced by α-TOS, preventing the formation of autophagosomes. α-TOS induced mitochondrial destabilization leading to the release of AIF. Translocation of AIF from mitochondria to the nucleus, a result of the combinatorial treatment, was mediated by PARP1 activation. The inhibition of AIF as well as of PARP1 efficiently attenuated apoptosis triggered by the drug combination. Using a mouse model of prostate cancer, the combination of α-TOS, VK3 and AA was more efficient in tumor suppression than when the drugs were given separately, without deleterious side effects.

**Conclusions/Significance:**

α-TOS, a mitochondria-targeting apoptotic agent, switches at sub-apoptotic doses from autophagy-dependent survival of cancer cells to their demise by promoting the induction of apoptosis. Given the grim prognosis for cancer patients, this finding is of potential clinical relevance.

## Introduction

Mitochondria have recently emerged as intriguing targets for anti-cancer drugs [1]–[3]. Vitamin K3 (VK3), a synthetic version of vitamin K, exhibits cytotoxic activity in cancer cells by causing mitochondria-dependent damage by way of redox cycling as well as selectively inhibiting DNA polymerase-γ, the key enzyme of mtDNA replication [3], [4]. To potentiate its anti-cancer effect, VK3 has been combined with the redox-active ascorbic acid (AA). Hydrogen peroxide generated by the VK3-AA combination has been reported to induce cell death in different types of cancer [5], [6].

VK3-AA-induced oxidative stress could be different in tumor cell and normal cell metabolism, and may allow manipulation designed to improve cancer therapy. However, in response to oxidative stress cells activate pathways that promote their survival and adaptation [7]. One such stress-response mechanism is autophagy [8], [9]. ROS can induce autophagy, which can contribute to caspase-independent cell death or, in contrast, autophagy can promote a protective role against ROS-mediated death [10], [11]. Therefore, the discovery of molecules that regulate autophagy may be of great significance in the development of drugs for the treatment of cancer.

Recently, sub-lethal concentrations of VK3 and AA, together with a sub-apoptotic dose of α-tocopheryl succinate (α-TOS), have been shown to efficiently induce prostate cancer cell death which was characterized by DNA fragmentation, lysosomal/mitochondrial perturbation, cytochrome *c* release, while lacking caspase activation [12]. Increasing evidence suggests that caspase-independent pathways play an important role in cancer death, and the apoptosis-inducing factor (AIF) is emerging as a central mediator of this process [13]–[16]. Relevant to this premise, activation of the DNA repair enzyme poly (ADP-ribose) polymerase-1 (PARP1) has been reported as essential for AIF release by a mechanism that involves Ca^2+^ influx into the cytosol [17]–[20].

Using sub-toxic doses of α-TOS with sub-toxic concentrations of VK3+AA previously identified [12], we evaluated the effect of α-TOS on ROS-mediated autophagy triggered by VK3 and AA. The mechanism(s) involved in the cell death induced by the agent combination was also investigated. We report that VK3 plus AA induced early ROS formation associated with induction of autophagy in response to oxidative stress. Inhibition of autophagy uncovered the protective role of VK3+AA-induced autophagy in PC3 cells. α-TOS was found to reduce VK3+AA-induced ROS formation abrogating the ROS triggers autophagosomes formation. However, ROS produced were sufficient to induce PARP1 activation, which results in the release of the pro-apoptotic factor AIF, promoting cell death manifested by hallmarks of apoptosis including chromatin condensation. The clinical relevance of this research is provided by substantial suppression of cancer in mice treated by the combination of the three agents.

## Results

### α-TOS inhibits autophagy triggered by ROS generated in response to VK3 and AA treatment

**Figure 1:**
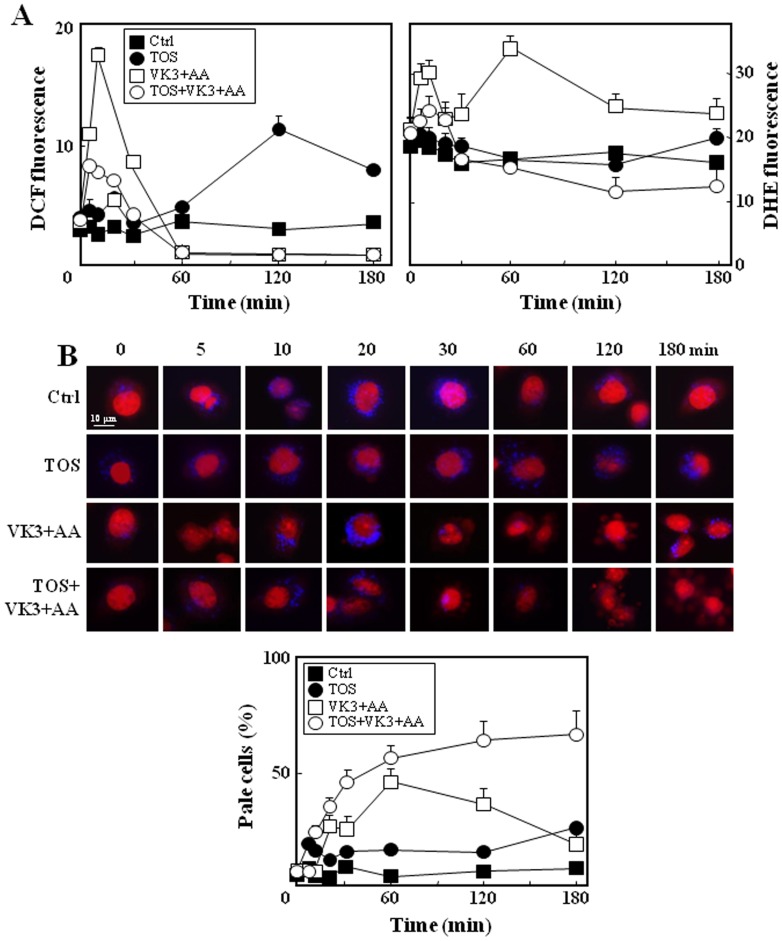
ROS generation in PC3 cell treated with á-TOS+VK3+AA. (A) PC3 cells were treated with á-TOS (30 µM), VK3 and AA (3 µM VK3, 0.4 mM AA) and assessed for ROS generation using DCF (an indicator of hydrogen peroxide) or DHE (an indicator of superoxide), expressed as mean fluorescence intensity. The cells were evaluated for the increased fluorescence using flow cytometry. (B) Microscopic visualization of DHE-stained PC3 cells following drug treatments over time. Upon interaction with superoxide, DHE (punctuate blue) yields ethidium bromide (EtBr) that accumulates in the nucleus and gives red fluorescence (upper panel). The nuclear blebs containing damaged chromatin reduce nuclear EtBr staining (pale cells). Flow cytometry quantification of pale cells (EtBr-negative cells, bottom panel) was performed in PC3 cells treated as indicated. The data shown are mean values ± S.D. (n = 3), the microscope images are representative of three independent experiments. Magnification 600×**,** scale bar  = 10 µm.

It has been reported that sub-toxic doses of the combination of VK3 (3 µM) with AA (0.4 mM) resulted in intracellular generation of ROS, but the cells died only in the presence of α-TOS (30 µM) [12]. To elucidate the role of oxidative stress in the combination treatment, ROS formation and cellular responses to oxidative stress (autophagy) were assessed over time. Treatment of PC3 cells with VK3 and AA resulted in the formation of peroxide-related molecules within 30 min, after which the ROS-associated fluorescent signal returned to its basal level. While α-TOS alone induced a late formation of peroxide-related molecules, which was observed after 120 min of incubation, it reduced the early increase in their levels in response to the treatment of the cells with VK3 and AA alone (Fig. 1A, left panel). To investigate if mitochondria are the major source of ROS generation, the levels of superoxide were assessed using dihydroetidium (DHE). Upon interaction with superoxide, DHE yields ethidium bromide (EtBr) that accumulates in the nucleus and yields red fluorescence [21]. Fig. 1A (right panel) documents increase superoxide levels within 20–60 min of treatment of the cells with VK3 and AA which was reduced in the presence of α-TOS. The DHE-derived EtBr nuclear staining was detected over time using fluorescence microscopy, and indicates that upon treatment with VK3 and AA, the apoptotic blebs containing nuclear components were formed at the cell surface after 60–120 min; 36–46% of cells were EtBr-negative (pale cells) as a consequence of chromatin loss. Reduced number of cells with nuclear blebs (18% of pale cells) was observed at prolonged incubation. The cells recovered after 180 min of incubation, thus indicating a reversible process. The presence of α-TOS markedly inhibited the ‘recovery’ of the blebs as evidenced by the increase of pale cells (66%), promoting cells death (Fig. 1B).

**Figure 2:**
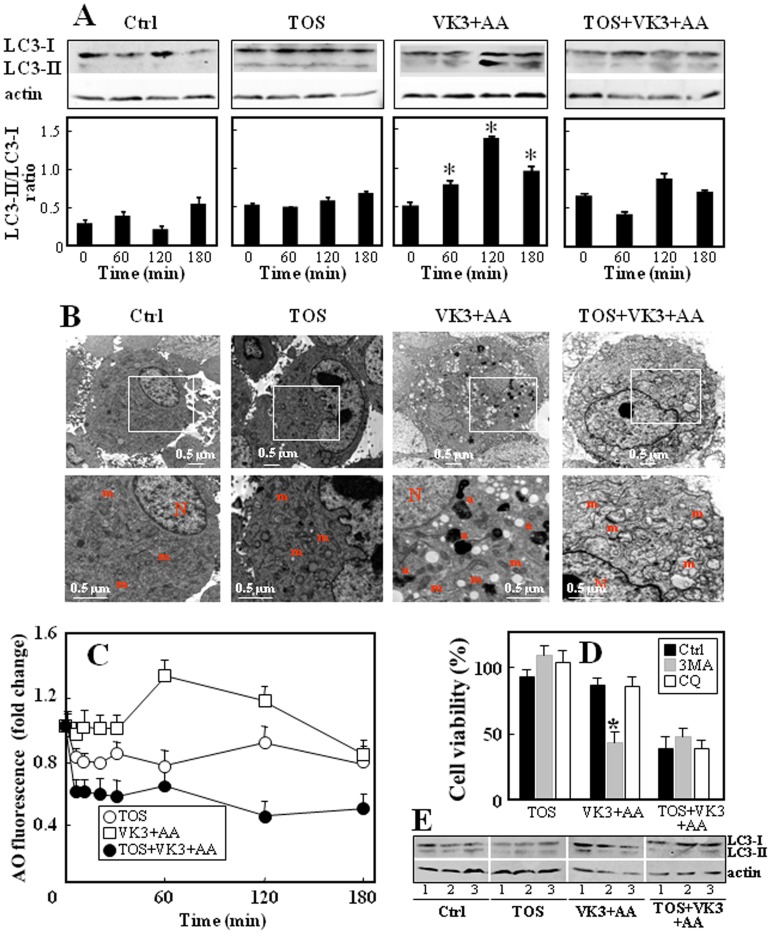
Effect of á-TOS on autophagy in PC3 cells treated with VK3+AA. (A) PC3 cells were treated with the drugs as indicated and the level of the LC3-II protein normalized to â-actin was expressed as mean ± S.D. of the LC3-II/LC3-I densitometry ratio. (B) PC3 cells were treated with the drugs as indicated for 2 h and assessed for morphology by TEM. Magnification 4,000×, scale bar  = 0.5 µm (upper images), magnification 9,000×, bar  = 0.5 µm; N =  nucleus, m =  mitochondria, a =  autophagosomes. (C) PC3 cells were treated as indicated, and formation of the AO-accumulating acidic vesicular organelles (orange-red fluorescence) was detected using flow cytometry. (D) PC3 cells were treated as indicated in presence or absence of autophagy inhibitors (3MA and CQ), and cell viability was assed after 24 h of incubation. (E) The formation of autophagosomes in control cells (1) and in the presence 3MA (2) and CQ (3) was evaluated by western blotting as the expression of LC3-II after 2 h of drug treatments. The data are expressed as a change with respect to untreated control, and are mean values ± S.D. (n = 3), the images are representative of three independent experiments. * p<0.05, compared with controls.

Having documented that the drug treatment induced generation of ROS, we next examined their effect on autophagy in PC3 cells by the evaluation of the expression pattern of the microtubule-associated protein-1 (LC3), by transmission electron microscopy (TEM), and via the formation of acidic vacuolar organelles [22]. LC3 exists in two forms, LC3-I (18-kDa) and LC3-II (16-kDa), the latter being membrane-bound and increased during autophagy by conversion of the former [23]. PC3 cells treated with VK3 and AA showed higher level of the LC3-II protein at 60–120 min post-treatment, which was inhibited in the presence of α-TOS (Fig. 2A). TEM was performed on PC3 cells following various treatment combinations. Control and α-TOS-treated cells featured mitochondria with well-defined cristae, intact double membrane, homogenous density and cylindrical shape. Appearance of a large number of autophagosomes with a double-membrane structure in the cytoplasm was observed by TEM after 120 min of treatment with VK3+AA, with little effect on the mitochondrial morphology. In contrast, when VK3 and AA were combined with α-TOS, prominent abnormal mitochondria with disorganized cristae, decreased electron density and increase in the minor axis consistent with mitochondrial swelling were observed, while formation of autophagosomes was not detected (Fig. 2B). The uptake of the red fluorescent AO further supports the inhibitory effect of α-TOS on the VK3+AA-induced autophagy (Fig. 2C).

Next we asked if autophagy affects cell death. To address this question, autophagy was suppressed with the pharmacological inhibitor 3MA (early phase inhibition) and CQ (late phase inhibition). PC3 cells were then treated with α-TOS or VK3+AA and α-TOS+VK3+AA. As shown in Fig. 2D, the inhibition of early autophagic process reduced cell viability in VK3+AA-treated cells in an extent comparable to what observed when α-TOS was combined with VK3+AA. This effect was not found when blocking the last step of the autophagic pathway. The formation of autophagosomes in the absence or presence of the autophagic inhibitors 3MA and CQ was evaluated as expression of LC3-II after drug treatments (2 h). The VK3+AA mixture induced LC3-II expression which was inhibited in presence of 3MA but not CQ (Fig. 2E). This indicates that autophagy is a protective response to VK3+AA-induced cell death, and the inhibition of autophagy at the early phase is a strategy to induce cell death.

### Mechanism of α-TOS, VK3 and AA-induced cell death

**Figure 3:**
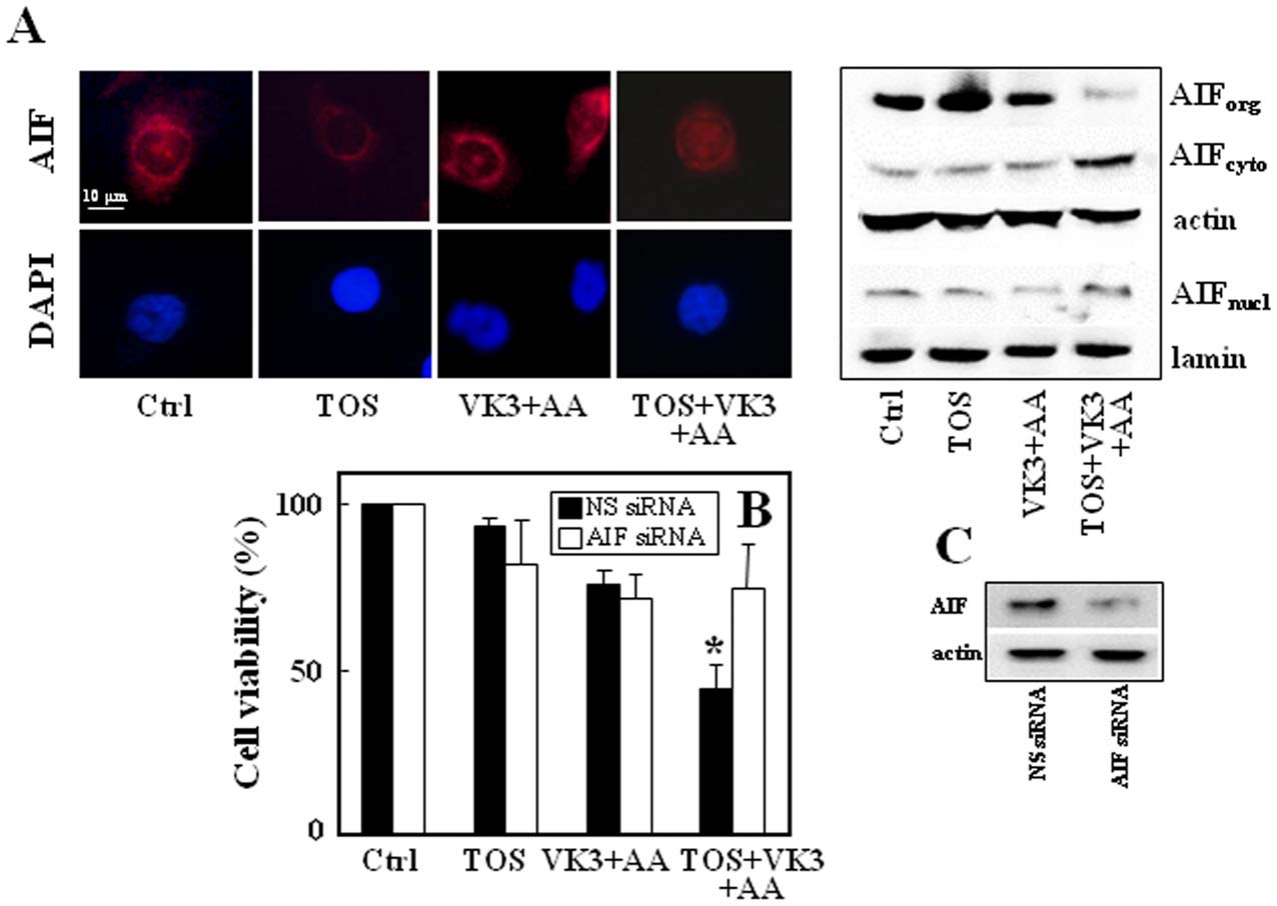
Treatment of PC3 cells with á-TOS+VK3+AA induces AIF translocation. Panel (A) shows fluorescence microscope images of AIF translocation to the nucleus after 180 min treatment with á-TOS (30 µM), VK3 and AA (3 µM VK3, 0.4 mM AA) alone or in combination (left panel). The nuclear translocation of AIF is demonstrated by similar distribution of the red (AIF-TRITC) and blue signal (nucleus); magnification 600×, scale bar  = 10 µm. The right panel shows western blot analysis of AIF translocation in cellular fractions. Organelle (org), cytoplasm (cyto) and nuclear (nucl) fractions were obtained from PC3 cells treated with the drugs as above and the level of AIF assessed. PC3 cells were transfected with NS siRNA or AIF siRNA, and assessed for AIF protein level (C). The transfected cells were treated as above for 24 h of incubation and assessed for viability using the MTT assay (B). The data shown are mean values ± S.D. (n = 3), the images are representative of three independent experiments. The symbol ‘*’ indicates statistically different results for AIF siRNA- and NS siRNA-transfected cells with p<0.05.

To investigate the mechanism(s) involved in cell death induced by the combination α-TOS with VK3+AA, we evaluated the role of AIF. Sub-cellular fractionation showed that only when α-TOS (30 µM) was combined with VK3 (3 µM) and AA (0.4 mM), AIF re-distributed from mitochondria to the cytoplasm and to the nucleus (Fig. 3A). To test whether AIF re-distribution is involved in cell death induced by the combination of the three agents, the protein was silenced and cell viability evaluated. As shown in Fig. 3B, siRNA targeting AIF efficiently silenced the AIF expression that, in turn, attenuated cell death induced by the combination of α-TOS, VK3 and AA. The efficacy of AIF siRNA is documented in Fig. 3C.

**Figure 4:**
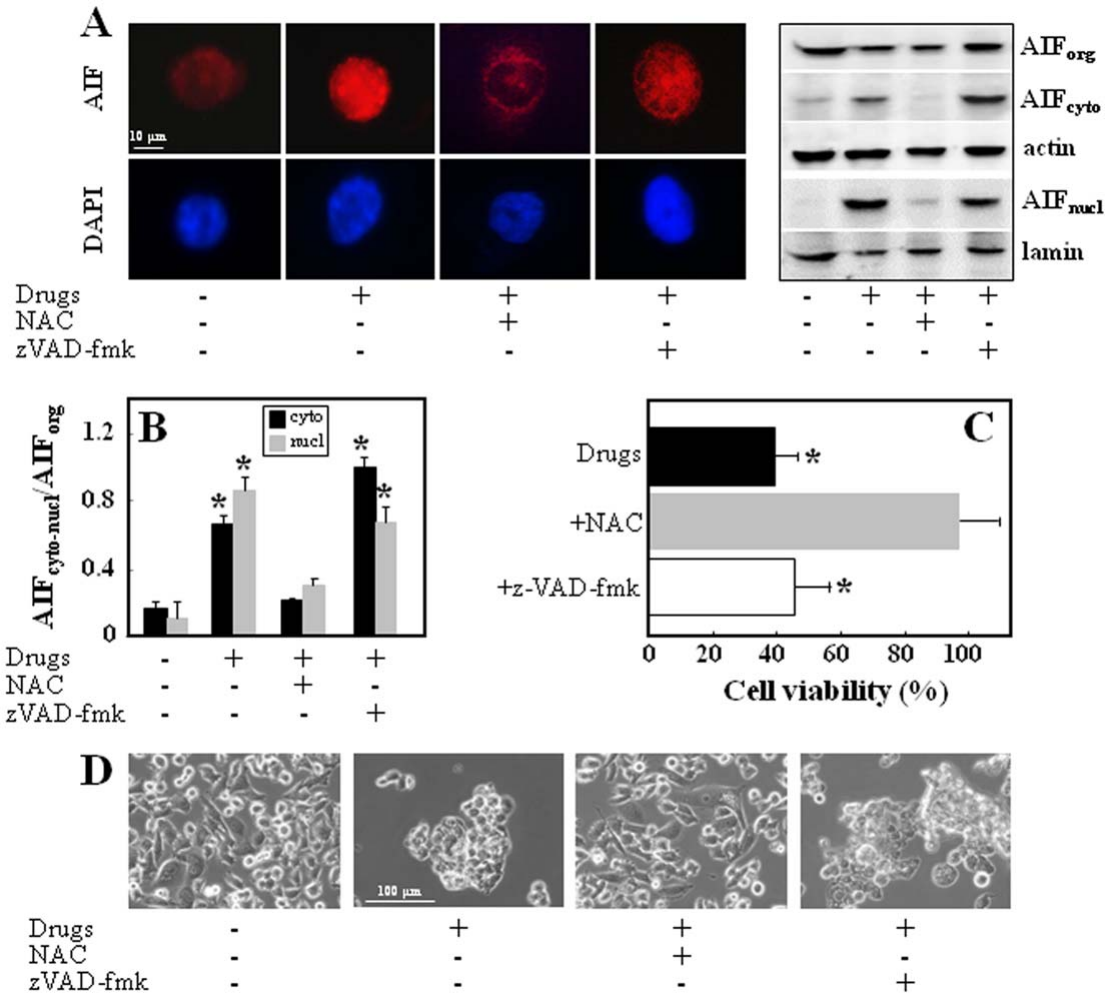
Treatment of PC3 cells with á-TOS+VK3+AA induces cell death via ROS-dependent and caspases-independent AIF translocation. Panel (A) documents AIF-TRITC visualized by fluorescence microscope, magnification 600×, scale bar  = 10 µm. (left panel), and western blot images (right panel) of AIF translocation to the cytosol and nucleus after180 min treatment with á-TOS (30 µM), VK3 and AA (3 µM VK3, 0.4 mM AA) in the absence or presence of NAC (10 mM) or z-VAD-fmk (10 µM). The AIF band density was normalized using â-actin or lamin, and is expressed as mean ± S.D. AIF_cyto_-AIF_nucl_/AIF_org_ densitometry ratio. (B). Cytotoxic effect of the combinatorial treatment on PC3 cells in the absence or presence of NAC (10 mM) and z-VAD-fmk (10 µM) was evaluated as cell viability after 24 h of exposure of the cells to the drugs (C), and phase contrast micrographs of the cell treated correspondingly are depicted (D); magnification 200×, scale bar  = 100 µm. The data shown are mean values ± S.D. (n = 3), the images are representative of three independent experiments. The symbol ‘*’ indicates statistically different results for untreated *vs* treated PC3 cells in the absence or presence of NAC or zVAD-fmk with p<0.05.

To determine the direct relationship between the increased intracellular ROS levels and AIF-mediated cell death induced by the combinatorial treatment, PC3 cells were pre-treated with the antioxidant NAC, which efficiently blocked cytoplasmic and nuclear translocation of AIF induced by the three agents, indicating the role of ROS (Fig. 4A,B). The inhibition of AIF nuclear translocation was associated with an abrogation of drugs-induced cell death (Fig. 4 C,D). Furthermore, to test whether the re-distribution of AIF occurs is a caspase-independent manner, the cells were exposed to the three agents in the presence of the pan-caspase inhibitor z-VAD-fmk, which neither blocked AIF translocation nor exerted any protective effect against the combinatorial treatment (Fig. 4 A–D).

**Figure 5:**
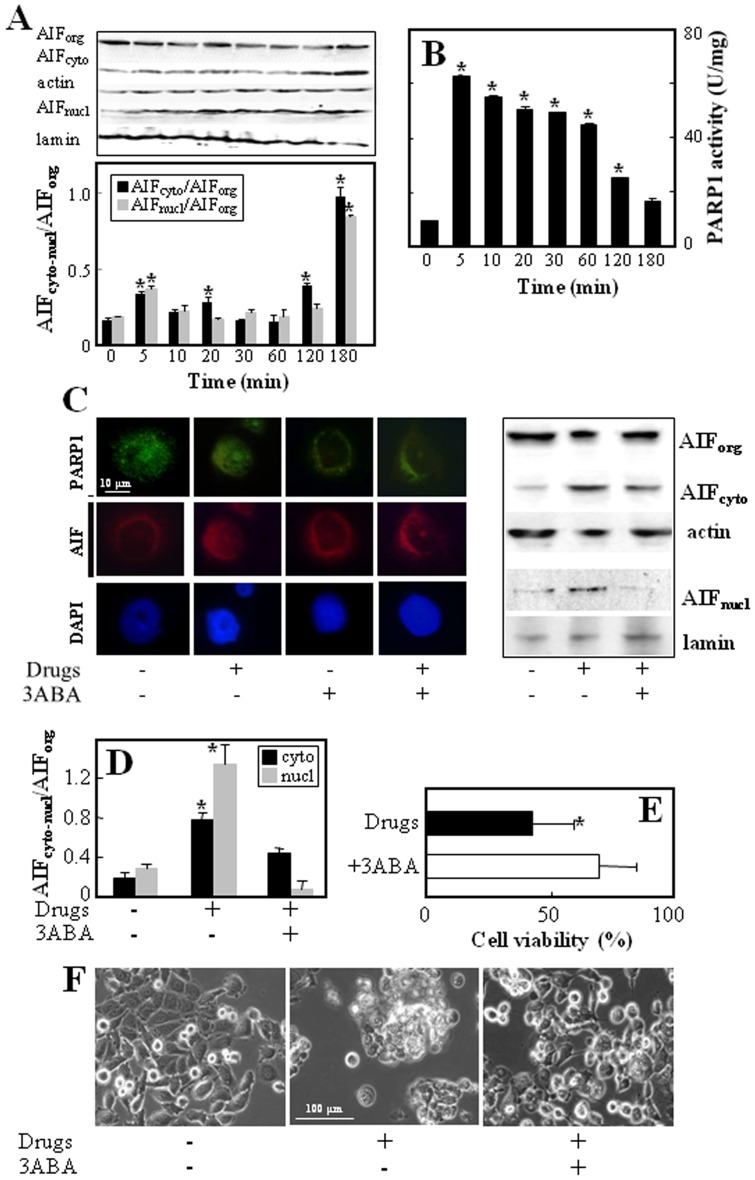
Treatment of PC3 cells with á-TOS+VK3+AA induces cell death via PARP1-dependent AIF translocation. Panel A shows kinetics of AIF release and panel B activity of PARP1 in PC3 cells exposed to α-TOS (30 µM), VK3 (3 µM) and AA (0.4 mM). Panel C shows fluorescence micrographs using PARP1-FITC and AIF-TRITC (magnification 600×, scale bar  = 10 µm) (left panel), and western blot images (right panel) of PARP1 and AIF translocation to the nucleus after 180 min treatment with á-TOS+VK3+AA in the absence or presence of 3ABA (2 mM). (D)The AIF band density was normalized to â-actin or lamin and expressed as mean ± S.D. of the AIF_cyto_-AIF_nucl_/AIF_org_ densitometry ratio. (E) Cytotoxic effect of the combinatorial treatment on PC3 cells in the absence or presence of 3ABA was evaluated as cell viability after 24 h of exposure of the cells to the drugs. Panel F shows phase contrast micrographs of the cells treated with the three drugs with or without 3ABA; magnification 200×, scale bar  = 100 µm. The data shown are mean values ± S.D. (n = 3), the images are representative of three independent experiments. The symbol ‘*’ indicates statistically different results for untreated vs treated PC3 cells in the absence or presence of 3-ABA with p<0.05.

**Figure 6:**
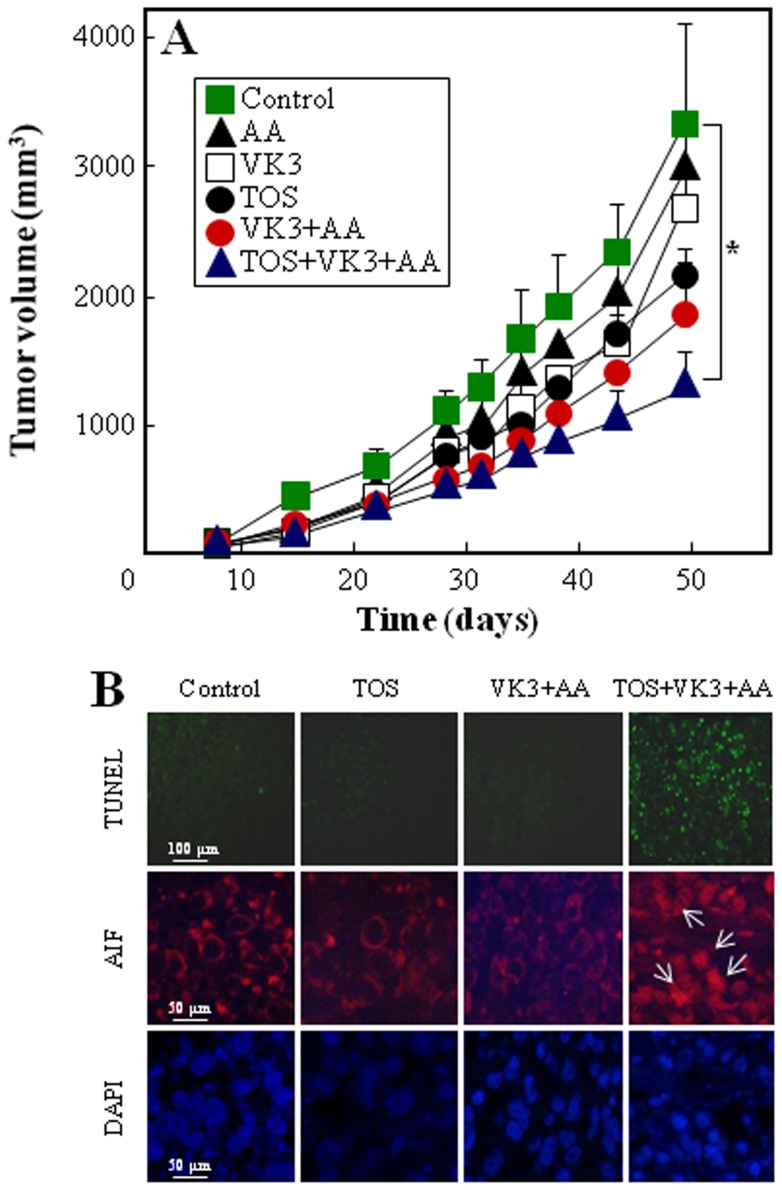
Treatment of PC3 cell-derived tumors with α-TOS+VK3+AA suppresses their progression. (A) Balb-c nude mice with xenografts derived PC3 cells were treated by oral administration of VK3 (3 mg/kg) andAA (400 mg/kg), and intraperitoneal injection of á-TOS (15 mg/kg) alone or in combination, as indicated, 5-times per week, and tumor volume (mm^3^) was quantified by calipers. (B) Formalin-fixed, paraffin-embedded tumor sections (5 µm) were inspected for TUNEL positivity (green punctuated cells) (magnification 200×, scale bar  = 100 µm) and were immuno-stained with anti-AIF IgG (white arrows indicated nuclear translocation of AIF-TRITC and pycnotic nuclei detected using DAPI); magnification 400×, scale bar  = 50 µm. Data of tumor volume are mean ± SEM (n = 8), no significant differences were found between control mice and single drug-treated mice, whereas significant differences were found between control mice and VK3+AA-treated animals on days 22–31 (p = 0.034) and α-TOS+VK3+AA-treated animals on days 43–49 (p = 0.043).

It has been previously reported that ROS-mediated DNA damage triggers activation of PARP1 and subsequent cell death [24]. PARP1 activation generates the PAR polymer in the nucleus and it translocate to mitochondria to mediate AIF release [20]. To clarify the relationship between AIF and PARP1, we evaluated AIF release and PARP1 activity in PC3 cells after exposure to α-TOS, VK3 and AA at different time points. AIF translocation to cytoplasm was detectable within 5 to 60 min of the combinatorial treatment of the cells, and cytoplasmic release of AIF was concomitant with PARP1 activation. In contrast, translocation of AIF to the nucleus was delayed by at least 60 min (Fig. 5A,B). Inhibition of PARP1 by 3ABA attenuated both AIF nuclear translocation (Fig. 5C,D) and cell death induction (Fig. 6 E,F).

### Treatment with α-TOS+VK3+AA suppresses tumor progression

In order to assess the anti-cancer effect of the three drugs, Balb-c nude mice with PC3 xenografts were orally treated with sub-toxic doses of VK3 (3 mg/kg), AA (400 mg/kg), and with α-TOS (15 mg/kg) delivered intraperitoneally, and with the combinations VK3+AA, or α-TOS+VK3+AA. A significant tumor growth inhibition was observed in the VK3 plus AA treatment group; this effect was considerably enhanced at prolonged times of treatment when the two agents were combined with α-TOS (Fig. 6A). The combinatorial therapy with the three drugs induced DNA strand break formation (TUNEL positivity), corresponding to an apoptotic process, which was associated with nuclear AIF translocation (Fig. 6B). Greater tumor cell death areas were found in mice exposed to the tri-combinatorial therapy with respect to the untreated mice (85±16×10^3^ µm^2^ *vs* 238±45×10^3^ µm^2^, respectively, *p*<0.05). This treatment appeared to exert no detectable side toxicity, since the mice showed no weight loss as well as no signs of cytotoxicity in the kidney and liver as detected by histological evaluation and by biochemical analyses (data not shown).

## Discussion

Oral administration of AA and VK3 was used in the treatment of prostate cancer patients [25]. This approach was beneficial when the drug combination was co-administrated with known chemotherapeutic agents [26]. Accordingly, co-administration of sub-lethal doses of AA+VK3 (redox-active system) with the redox-silent α-TOS resulted in efficient *in vitro* cell death. The combination of VK3+AA was shown to synergistically promote production of hydrogen peroxide in the extracellular milieu [12], [27].

Consistent with the previous observations, we found that the combination of VK3+AA caused fast generation of both hydrogen peroxide (DCF fluorescence) and superoxide (DHE fluorescence), peaking at 10–20 min and 30–60 min of exposure of the cells to the two drugs (*cf* Fig. 1A). In response to this oxidative stress, the cells activated the autophagic pathway. This was evidenced by the formation of nuclear blebs and autophagic vesicles (acidic vacuolar organelles), as well as increased levels of the LC3-II protein (*cf* Fig. 2A–C). However, after 180 min of this treatment, the cells completely recovered. This is shown by DHE oxidation-induced EtBr nuclear staining, which documents that the increased index of pale cells observed after 60 min of treatment with VK3+AA markedly decreased over prolonged time periods (*cf* Fig. 1B). The autophagy inhibitor 3MA (early autophagic process) triggers the VK3+AA-induced cell death indicating that autophagy is a protective mechanism in the context of PC3 cells (*cf* Fig. 2D). These findings indicate that the early oxidative stress results in an adaptive response of the cancer cells that allows for their recovery from the insult. On the other hand, sub-apoptotic doses of α-TOS induced a reduction in ROS formation triggered by VK3+AA (*cf* Fig. 1A). We can hypothesize that the hydrolysis of α-TOS by esterases in cells leads to generation of α-tocopherol (α-TOH), which acts as an ROS scavenger [28]. As previously found [29], we observed that the ROS generated in response to α-TOS mainly consist of peroxide-related species, but not superoxide. In contrast, α-TOS was reported to induce superoxide production via interaction with complex II of the mitochondrial respiratory chain [30]. We cannot exclude that in our experimental conditions, the sub-toxic doses ofα-TOS lead to the generation of low amounts of superoxide, which may be rapidly dismutated and escape detection.

No formation of autophagosomes or altered morphology of organelles was observed in α-TOS- and VK3+AA treated cells (*cf* Fig. 2A–C). This links the reduction of the ‘adaptive’ early oxidation stress with the prevention of the autophagic process induced by the combination of VK3+AA alone, and is further corroborated by TEM, revealing the absence of autophagosomes in cells treated by the combination of the three drugs (*cf* Fig. 2B). Inhibition of autophagy induced byα-TOS promoted appearance of an increased sub-set of pale cells, formation of nuclear blebs and mitochondrial swelling with disorganized cristae and decreased electron density, all features of cell death. Therefore, inclusion of α-TOS in the combinatorial treatment of PC3 cells inhibits the adaptive mechanisms and survival leading to the induction of their death.

Induction of autophagy has been reported to promote survival of tumor cells, thereby counteracting or limiting the efficacy of cell death induction by chemotherapeutic agents, jeopardizing the outcome of cancer therapy [31]. In agreement with this notion, our data show a protective role of autophagy upon exposure of PC3 cells to VK3 and AA. We found that the treated cells, which exhibited morphology reminiscent of (autophagic) cell death, largely recovered, and only a minor fraction of the cells with disrupted mitochondrial trans-membrane potential was beyond rescue and died under these conditions.

It was found earlier that apoptosis is delayed in cells lacking the Bax and Bak proteins that are required for mitochondrial outer membrane permeabilization (MOMP), which is a hallmark of apoptotic cell death and a prerequisite for the cell to cross the ‘point of no return’ [32]. It was found that α-TOS induces translocation of Bax into mitochondria in breast cancer cells [33]–[36]. Another report documented α-TOS to induce mitochondrial apoptosis involving the FoxO1-Noxa-Bak axis [37], [38]. An AIF-mediated caspase-independent intrinsic pathway via Bak has been previously described [39]. The AIF soluble fragment is released from mitochondria upon outer membrane permeabilization through Bax/Bak pores [40]. We show that AIF release from mitochondria is necessary for cell death induced by the combinatorial treatment of prostate cancer cells with α-TOS+VK3+AA at sub-toxic doses of the individual agents. Silencing the AIF protein by siRNA substantially attenuated the death of PC3 cells induced by the combinatorial treatment (*cf* Fig. 3).

ROS production plays a key role in the release of AIF and the subsequent promotion of cell death. We document that this premise holds for our system, showing that the ROS scavenger NAC completely abrogated death of PC3 cells exposed to the combination of α-TOS+VK3+AA (*cf* Fig. 4). We further document that ROS-dependent PARP1 activation is associated with the release of AIF from mitochondria to the nucleus (*cf* Fig. 5). In response to DNA damage, PARP1 catalyzes the synthesis and transfer of PAR units to the acceptor proteins, whereby modulating their activities and regulating DNA repair [41], [42]. However, excessive DNA damage activates a unique PARP1-dependent cell death program, which is caspase-independent but dependent on AIF [43]. Even if to a lesser extent with respect to VK3+AA, the α-TOS+VK3+AA combination induced early ROS formation, which was previously found to induce early DNA damage [12]. The ROS-induced DNA damage triggered a robust increase in the PARP1 activity, which resulted in the release of AIF from mitochondria to the cytoplasm, and further to the nucleus. Thus, as s a consequence of PARP1 activation, the AIF protein translocated from the cytoplasm to the nucleus, resulting in the induction of chromatin condensation with ensuing cell death (*cf* Fig. 5).

Others have observed that high doses of AA induced autophagy in prostate cancer cells, which did not recover and were committed to die [10], [44]. Different doses of the drugs exert different effects on cells by producing varying amounts of ROS. Based on the ROS insult, the cells differently respond by the induction of their survival or death program. Here, sub-toxic doses of VK3+AA induced an ROS insult not able to trigger cell death, but causing damage of cellular organelles with subsequent activation of the survival autophagic process. The presence of α-TOS reduced the VK3+AA-induced oxidative insult, thus inhibiting the cellular survival response. However, the ROS formed were sufficient to induce PARP1 activation resulting in AIF release and the subsequent cell death.

Given the possible anti-cancer activity of the three agents, the effect of the combination of α-TOS+VK3+AA on tumor growth was examined in nude mice. Oral administration of the mixture of VK3+AA significantly reduced tumor growth on days 22–31 (p = 0.034). No TUNEL positivity was found in sections of the VK3+AA-treated tumors, suggesting that the reduction in tumor size induced by VK3+AA was due to tumor growth inhibition rather than cell death (*cf* Fig 6A). This is corroborated by the fact that at prolonged time of drug exposure (days 35–49) the tumors re-started to grow. Treatment with α-TOS+VK3+AA significantly inhibited tumor growth (days 43–49, p = 0.04), inducing wide areas of cell death associated with AIF nuclear translocation compared to the control animals and those treated with the individual drugs (*cf* Fig. 6A,B). Notably, the anti-tumor effect of the α-TOS+VK3+AA combination was associated with no signs of secondary deleterious effects. These findings support the *in vitro* data showing that α-TOS inhibits cancer cell recovery and survival following the VK3+AA treatment, inducing efficient cell death at prolonged times of exposure to the combination of the three agents.

## Conclusions

**Figure 7:**
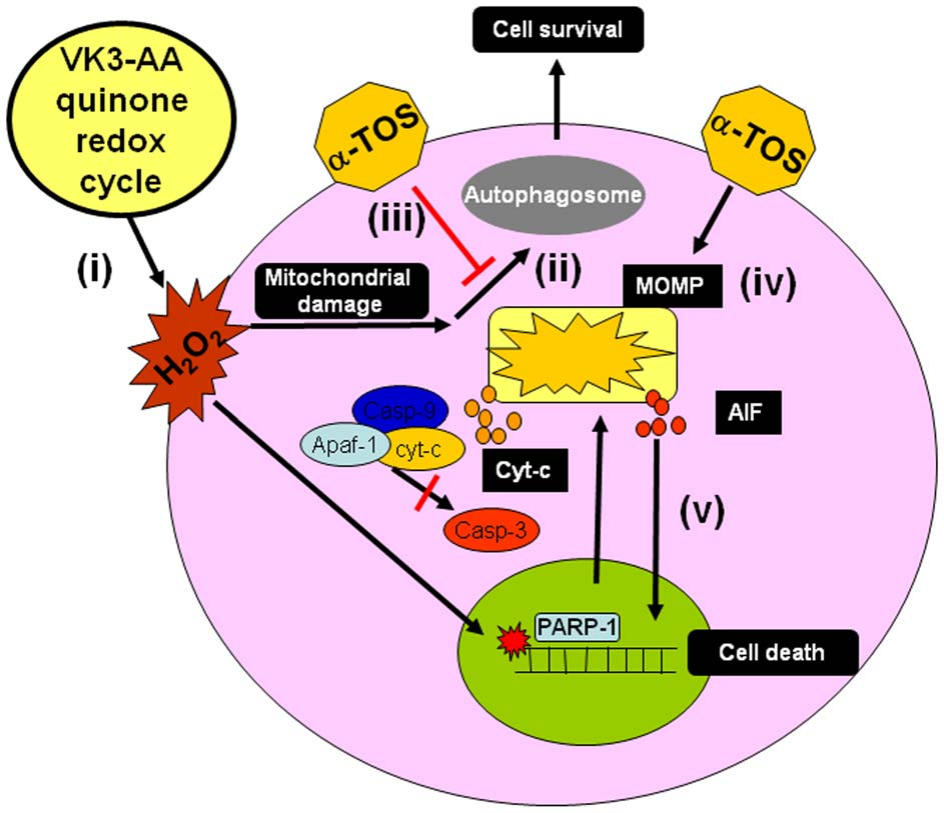
A model depicting the interrelationship of the various events in drug-induced AIF release, autophagy, and induction of cell death. The VK3+AA combination induces ROS formation (i), to which the cells response by autophagy to eliminate damaged mitochondria and other organelles (ii). α-TOS inhibits the survival process of autophagy reducing the VK3+AA-induced oxidative stress (iii), leading to caspase-independent cell death involving PARP1-dependent AIF release (iv).

Many factors such as tumor vascularization and cancer cell uptake affect drug concentrations within tumor mass. Therefore, at tumor level, the anti-cancer drugs could be not at the killing level even when administrated at high concentrations. Thus, instead to induce cell death, low levels of anti-cancer drugs could induce an adaptive response leading the cancer cells to proliferate. Here, we found that the adaptive cellular response to oxidative stress induced by the redox-active system of VK3+AA was overcome by the combination with the redox-silent agent α-TOS, which caused a switch from autophagic survival to cell death (Fig. 7). Since the serum levels of the anti-cancer agents were below the detection limit, their pharmacokinetics was not evaluated, therefore the effective concentration of the agents that could translate into clinical settings are not precisely known. In clinical practice, low doses of AA and VK3 can be easily achieved by oral administration. Since α-TOS completely converts to the inefficient α-tocopherol after oral application, intravenous or transdermal administration represents a plausible way to achieve its pharmacological levels and the anti-cancer effect. Notwithstanding the clinical means of administration, which is yet to be tested, this report demonstrates the potential use of targeted drug combination in the treatment of prostate cancer.

## Materials and Methods

### Cell culture and treatment

The prostate cancer cell line PC3 was purchased from the ATCC and grown in the RPMI-1640 medium supplemented with 2 mM L-glutamine, 100 U/ml penicillin, 100 µg/ml streptomycin and 10% FBS, at 37°C and 5% CO_2_ in humidified atmosphere. AA (Sigma, St Louis, MO, USA) was freshly prepared in PBS (pH 7.4) and immediately used at the final concentration of 0.4 mM. α-TOS and VK3 (menadione) (both from Sigma) were dissolved in ethanol and DMSO, respectively, diluted in the complete medium to the final concentration of 30 µM and 3 µM, respectively, and added to cells at 0.1% of the solvent. The ROS scavenger N-acetyl-L-cysteine (NAC), the PARP1 inhibitor 3-aminobenzamide (3ABA), the pan-caspase inhibitor z-VAD-fmk, the autophagy inhibitors 3-methyladenine (3MA) and chloroquine (CQ) (all from Sigma) were added to the culture medium 2 h before treatments at 10 mM, 2 mM, 10 µM, 0.5 mM and 10 µM, respectively.

### Transfection with small interfering RNA (siRNA)

Two siRNA oligonucleotides (GGA GGU CGA AUG GGU AAA GGA GCA TdT and ACG AUA UAA AGU UGG GAA GGA GGC GdT) were used to silence AIF protein expression. As a negative control, non-silencing RNA (NS RNA) (CCA CTA CCT GAG CAC CCA GdT) was used. Both siRNAs and nsRNA were obtained from Integrated DNA Technology (Tema Ricerca, Bologna, Italy). For transfection, PC3 cells were seeded in 96-well plates at 2×10^4^ per well and transfected with AIF siRNAs and nsRNA (0.1 µg/well) using the TransIT-LT1 transfection reagent (Mirus, Tema Ricerca, Bologna, Italy) according to the manufacturer's instructions.

### Immunocytochemistry

The parental or AIF-silenced PC3 cells were seeded on cover-slips in 6-well plates. The cells were allowed to attach overnight and then incubated for 1 h with α-TOS or the drug combination (VK3 and AA, or α-TOS, VK3 and AA). The cells were then washed with PBS, fixed with freshly prepared 4% formaldehyde in PBS, and incubated with saponine solution (0.05% saponine and 2% FCS in PBS), after which they were incubated with rabbit anti-AIF IgG (Cell Signaling Technology, Beverly, MA) or rabbit anti-PARP-1 (clone C2-10, Trevigen, Gaithersburg, MD, USA) in the saponine solution at 4°C, followed by TRITC-conjugated and FITC-conjugated, anti-rabbit secondary IgG (Sigma), respectively. Cover-slips were mounted with Vectashield (Vector Laboratories, Burlingame, CA, USA) and examined under a fluorescence microscope (Zeiss, Axiocam MRc5; magnification 600 x).

### Cell viability assay

Cell viability was evaluated by the MTT method. PC3 cells (both parental and AIF-silenced) were plated in 96-well flat-bottom plates at the density of 2×10^4^ per well, allowed to attach overnight, and treated with α-TOS or the drug combination (VK3 and AA, or α-TOS, VK3 and AA) in the presence or absence of NAC (10 mM), z-VAD-fmk (10 µM), 3-ABA (2 mM), 3-MA (0.5 mM) or CQ (10 µM). After 24 h, 10 µl (3–4,5-dimethylthiazol-2-yl)-2,5-diphenyltetrazolium bromide (MTT; Sigma) were added to each well and the plate incubated at 37°C for 3 h. After removing the media, 200 µl of isopropanol were added to dissolve the crystals. Absorbance was read at 550 nm in an ELISA plate reader (Sunrise, Tecan, Milan, Italy), and the results expressed as relative change with respect to the controls set as 100%.

### Subcellular fractionation and western blot analysis

PC3 cells (3×10^5^ per well in 6-well plates) were treated with α-TOS, VK3 and AA for 180 min, harvested, and the pellet re-suspended in the digitonin cell permebilization buffer (Trevigen, Gaithersburg, MD). The supernatant containing the cytosolic fraction was collected. The remaining pellet (organelle fraction) was lysed in the RIPA buffer (20 mM Tris-HCl, pH 7.5, 150 mM NaCl, 1 mM Na2EDTA, 1 mM EGTA, 1% NP-40, 1% sodium deoxycholate, 2.5 mM sodium pyrophosphate, 1 mM β-glycerophosphate, 1 mM Na_3_VO_4_,1 µg/ml protease inhibitors). For preparation of the nuclear fraction, the cells were washed twice with ice-cold PBS and re-suspended in PBS containing 10 mM NaPO_3_, 150 mM NaCl, 0.5% Triton X-100, pH 7.4, supplemented with protease inhibitors. After 20 strokes in a Dounce homogenizer, the unbroken cells were spun down at 2,500×g for 15 min at 4°C. The nuclear fraction was re-suspended in ice-cold hypertonic buffer (10 mM NaPO_3_, 350 mM NaCl, pH 7.4), mixed and sonicated on ice (5×20 s). After centrifugation at 2,500×g for 15 min at 4°C, the supernatant was collected.

For western blot analysis, protein samples (50 µg per lane) were resolved using 12.5% SDS-PAGE, and transferred to nitrocellulose membranes, and incubated overnight with anti-AIF, or anti-LC3 IgG (Cell Signaling Technology). β-Actin and lamin (Bethyl, Montgomery, TX, USA) were used as loading controls for the cytosolic and nuclear fractions, respectively. After incubation with an HRP-conjugated secondary IgG (Sigma), the blots were developed using the ECL detection system (Pierce Biotechnology, Rockford, IL, USA). Band intensities were visualized by ChemiDoc using the Quantity One software (BioRad).

### Assessment of PARP activity

PARP activity was evaluated using a colorimetric assay kit (Trevigen, Gaithersburg, MD) based on the incorporation of biotinylated ADP-ribose into histone proteins. Cell and tissue lysates containing 50 µg proteins were loaded into 96-well plates coated with histone and biotinylated poly-ADP-ribose (PAR), allowed to incubate for 1 h, treated with streptavidin-horseradish peroxidase (HRP), and read at 450 nm in an ELISA plate reader. The PARP activity was expressed as PARP unit/mg of protein.

### Assessment of ROS generation

Intracellular hydrogen peroxide and superoxide anion levels were estimated using the fluorescent dye 2′7′-dichlorofluorescein diacetate (DCFDA; oxidized by hydrogen peroxide to DCF) and dihydroethidine (DHE; oxidized by superoxide to ethidium bromide), respectively. PC3 cells (10^5^) were seeded in 24-well plates, treated with the drugs as above, and supplemented with 20 µM DCFDA or DHE per well. After 30 min the florescent probe was removed, the cells collected, washed and resuspended in PBS, and analyzed by flow cytometry (FACS Calibur, Becton Dickinson). The level of ROS was expressed as mean fluorescence intensity. Cells labelled on cover slips were examined using fluorescent microscopy (magnification 600×).

### Detection of acid vesicular organelles

PC3 cells were plated in 6-well plates at 2×10^5^ per well. After exposure to α-TOS, VK3 and AA, the cells were incubated with 5 µg/ml acridine orange (AO) for 15 min. Floating and attached cells were collected, re-suspended in PBS, and red fluorescence evaluated by flow cytometry. The level of AO was quantified as mean fluorescence intensity and expressed as fold change with respect to the control.

### Transmission electron microscopy (TEM)

PC3 cells were plated overnight in 6-well plates at 2×10^5^ per well. After treatment with the drugs as mentioned above, the cells were harvested, fixed in 2% glutaraldehyde for 4 h, and centrifuged to form pellets. The pellets were rinsed in 0.1 M cacodylate buffer (Electron Microscopy Sciences, Hatfield, PA, USA), post-fixed in 1% osmium tetroxide for 60 min at 4°C, dehydrated in acetone, and embedded in an Epon-Araldite mixture. Thin sections were obtained with a Reichert Ultratome (Reichert Technologies, Depew, NY, USA), stained with lead citrate, and examined using the Philips CM 10 transmission electron microscope (Philips, Eindhoven, The Netherlands).

### Ethics statement

All procedures and experiments were carried out in the Angelini animal facility according to the European Communities Council Directive (609/86 EEC), the Italian law for the care and use of experimental animals (DL 116/92) and approved by the Italian Ministry of Health by decree # 46/2010-B, released on March 9, 2010. All experiments respected the welfare of animals and have been conducted strictly according to the legal and ethical requirements demanded by law.

### Mouse tumor experiments

Balb-c nude mice (5–6 weeks old) obtained from Harlan Laboratories were used for subcutaneous (s.c.) tumor implantation. Xenografts were established by s.c. injection of PC3 cells (10^6^) in Matrigel (200 µl). Following inoculation, animals were checked for palpable tumor presence and general clinical conditions. Based on previous *in vivo* studies, α-TOS [45], VK3 [46] and AA [47] the doses of the drugs were considerably reduced to obtain sub-toxic doses. At day 7 after cancer cell implantation, mice were orally treated with AA dissolved in sterile water (400 mg/kg) or VK3 dissolved in DMSO and then diluted in PBS at 3 mg/kg, and with α-TOS dissolved in ethanol, then diluted in PBS at 15 mg/kg administered intraperitoneally. Five treatment groups were established, the AA group, the VK3 group the α-TOS group, the VK3 and AA group, and the α-TOS,VK3 and AA group (8 mice per group). Drugs were administrated daily, five days per week. PC3-injected mice receiving vehicle were used as positive controls. Body weight was assessed and, when possible, tumor width (*w*) and length (*l*) were measured by calipers twice a week. Tumor volume (*v*), in mm^3^, was calculated using the following formula: v =  (*w*)^2^ × *l/*2. Based on tumor volume, animals were sacrificed 49 days following cancer cells inoculum. To evaluate the cytotoxicity of the agents to normal tissues, biochemical parameters, kidney and liver histological evaluation were performed. Blood was collected in the presence of heparin by intracardiac puncture for biochemical analysis. Tumors, kidneys and livers were harvested from animals perfused with PBS and 4% formalin using a peristaltic pump, placed in fresh 10% neutral-buffered formalin and embedded in paraffin for further evaluation. Tissue sections (5 µm) were stained with hematoxilin-eosin or immunostained with anti-AIF, or were subjected to the TUNEL assay (TACS-XL-Blue, Trevigen, Gaithersburg, MD, USA), and viewed in an optical microscope (Zeiss, Axiocam MRc5; magnification 25× or 600×). The tumor cell death was quantified evaluating cell death areas (n = 10) of tumor sections and expressed as mean ± S.D. area values (µm^2^) using the Axionvision Release 4.6 software.

### Statistical analysis

All experiments were conducted at least thrice, and data are shown as mean ± SD. The animal data were expressed as mean ± SEM. Multiple comparisons were evaluated by Kruskal-Wallis analysis, the differences between groups were assessed using the Mann-Whitney-U-test. For the in vivo study, the differences among groups were assessed by ANOVA with repeated measure followed by Tukey HSD post-hoc test. The data were considered statistically significant at p<0.05. All data generated in this study were analyzed using the SPSS software.
